# Research progress, challenges and perspectives on PNPLA3 and its variants in Liver Diseases

**DOI:** 10.7150/jca.57951

**Published:** 2021-08-13

**Authors:** Hongjiao Xiang, Zecheng Wu, Junmin Wang, Tao Wu

**Affiliations:** Institute of Interdisciplinary Integrative Medicine Research, Shanghai University of Traditional Chinese Medicine, Shanghai 201203, China.

**Keywords:** PNPLA3, NAFLD, type 2 diabetes, liver fibrosis, hepatocellular carcinoma

## Abstract

The human patatin-like phospholipase domain-containing 3 gene (PNPLA3) is highly expressed in liver and adipose tissue and encodes a transmembrane polypeptide chain containing 481 amino acids. The I148M variant of PNPLA3 is a single nucleotide polymorphism, which is related to a variety of liver and cardiovascular diseases and their complications (such as non-alcoholic fatty liver disease, liver fibrosis, coronary artery disease). This review mainly describes the pathophysiological effects of PNPLA3 and its variants, and their roles in the progression of liver disease and its complications.

## Introduction

Liver diseases have a significant impact on people's quality of life. Deaths caused by cirrhosis and liver cancer account for 3.5% of the global mortality rate 1. The occurrence of liver disease has a complicated relationship with environmental and genetic factors. Patatin-like phospholipase domain-containing 3 gene (PNPLA3) and its variants (PNPLA3 I148M) have been confirmed to be closely related to the occurrence of liver disease. The human PNPLA3 is a protein that contains a patatin-like phospholipase domain and has nine types 2. Patatin, as the main protein of potato tuber, has nonspecific lipoacyl hydrolase activity 3, 4. In 2001, Baulande et al. 5 identified genes that participated in the reprogramming of gene expression during the adipose differentiation of the murine 3T3 preadipocyte cell line, and it was then that *PNPLA3* was first identified and cloned.

**Figure 1:**
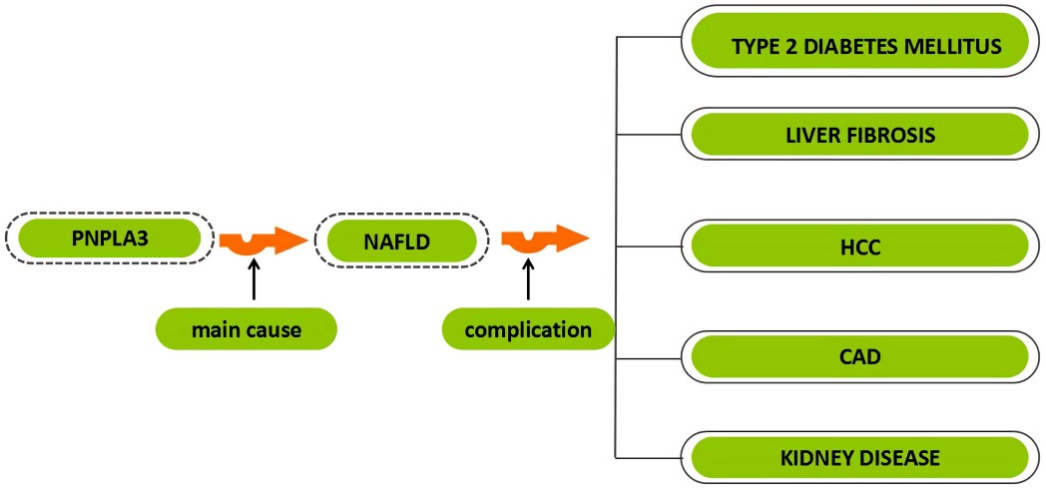
** The relationship between PNPLA3 and different diseases.** PNPLA3, patatin-like phospholipase domain-containing 3 gene; NAFLD, non-alcoholic fatty liver disease; HCC, hepatocellular carcinoma; CAD, coronary artery disease.

This article reviews the discovery and role shaping research progress and challenges about PNPLA3, and mainly elaborates the role of a non-synonymous variant of PNPLA3 (rs738409, I148M) in liver disease and related diseases. We also summarized the discovery and history of knowledge about PNPLA3 (Figure 1, Ttable 1).

## Structure of PNPLA3

The *PNPLA3* gene encodes a protein of 481 amino acids 6, and it is highly expressed in liver and adipose tissues. In humans, PNPLA3 gene is located on the long arm (q) and (22q. 13.31) of chromosome 22. Its molecular weight is predicted to be 52.8 kDa 5. It has 9 exons and a transcription length of 2805 base pairs 7. The N-terminal of PNPLA3 contains a patatin like domain with the common sequence of ser-Asp catalytic dimer (Gly-X-Ser-X-Gly and Asp-X-Gly/Ala) 8. The non-synonymous variation of PNPLA3 (rs738409, I148M) is caused by the substitution of isoleucine (I) with methionine (M) in the amino acid coding sequence 148 (I148M). PNPLA3 I148M has received widespread attention due to its high correlation with the risk of non-alcoholic fatty liver disease (NAFLD) 9, 10. The wild-type PNPLA3 is mainly distributed between membrane and lipid droplets, and closely bound with membrane. The allocation and positioning of PNPLA3 I148M have not changed significantly 11, 12.

## Physiological function of PNPLA3

The PNPLA3 protein is also called adiponutrin. In the liver, it is mainly found in hepatocytes 13, stellate cells 14, 15 and sinusoidal cells 16. PNPLA3 levels in stellate cells were lower than stellate hepatocytes 17. Through biochemical grading studies, it was found that PNPLA3 is mostly present in lipid droplets in liver cells 11, and PNPLA3 I148M has relation to droplets of larger size with reduced triglyceride (TG) hydrolysis, as shown by recent studies 18, 19. Thus, PNPLA3 protein is mainly connected with lipid droplets of hepatocytes.

In terms of the role of PNPLA3, it was thought to help regulated the development of adipocytes and the production and breakdown of fats in hepatocytes and adipocytes, through the processes known as lipogenesis and lipolysis. PNPLA3 protein has lipase activity and can hydrolyze triglycerides. This lipase activity is mainly for monounsaturated fatty acids and polyunsaturated fatty acids 12, 20. Mitsche et al. found that the transfer of vLCPUFAs from TG to phospholipids (PLs) in the liver requires the participation in PNPLA3. This effect was confirmed in PNPLA3 inactivated mice and PNPLA3^-/-^ mice 21. On the other hand, PNPLA3 also participates in the remodeling of TG and PL in the form of vLCPUFA-specific TG hydrolase 21. Chen et al. 22 indicated that PNPLA3 mRNA levels decreased after fasting and that PNPLA3 expression increased with refeeding of mice by both insulin and glucose 23. It was also shown that the PNPLA3 was used to help process and store fats in the diet. In the liver, levels of PNPLA3 were positively correlated with body mass index (BMI) 24. However, some studies have found that after knocking out PNPLA3, the TG content and TG hydrolysis in fat and liver tissues did not change significantly 25, 26. At the same time, PNPLA3 also had lysophosphatidic acid acyltransferase activity (LPAAT) 12. In some studies, it was mentioned that the change of LPAAT activity of PNPLA3 was related to the accumulation of TG. They proposed that the change of TG in lipid droplets was not related to its lipase activity, but was mainly determined by its LPAAT activity 27. BasuRay et al. 28 denied that the accumulation of TG was related to the activity of LPAAT. They proposed that PNPLA3 regulated the accumulation of TG in lipid droplets (LDs) through ubiquitination. From the above studies, it was found that the mechanism of PNPLA3 regulating TG was complicated. In other studies, it was also found that PNPLA3 also had retinyl esterase activity and participates in the metabolism of vitamin A in the body 29, 30. In addition, Huang et al. 17 revealed that PNPLA3 achieves nutritional control through a feed-forward loop, which started from transcriptional upregulation and expands through coordinated inhibition of protein degradation.

## PNPLA3 and liver disease

### PNPLA3 and NAFLD

The occurrence of NAFLD is related to many factors, mainly manifested as the accumulation of TG in liver cells and symptoms related to metabolic syndrome 31. NAFLD initially manifests as liver steatosis, which may develop into non-alcoholic steatohepatitis (NASH). It has the following characteristics: hepatocyte damage, hepatocyte swelling ('ballooning'), inflammatory infiltrate and the early development of perisinusoidal ('chicken wire') fibrosis. If the damage to the liver continues to increase, there is a high probability of causing liver cirrhosis, liver decompensation, liver cancer, etc. 32.

A study using PNPLA3-KO mice found that when endoplasmic reticulum stresses occured, PNPLA3 participated in liver fatty acid metabolism and triglyceride accumulation 33. Another study found that when mice were given a high-fat diet, the level of PNPLA3 in the liver increased significantly, suggesting that PNPLA3 may be involved in lipid metabolism under conditions of excess lipids 34. But most studies have shown that the occurrence of NAFLD is related to rs738409. Romeo et al. 35 found a non-synonymous variant of PNPLA3 (rs738409, I148M) in a 2008 study, which was a major risk factor for the development of NAFLD. They suggested that the incidence of PNPLA3 I148M in the Hispanic population was higher than that of European Americans and African Americans 35. Hotta et al. 36 genotyped 253 NAFLD patients and 578 control subjects using TaqMan analysis. They found that individuals with a G-allele of rs738409 were more likely to develop NAFLD in the Japanese population. Moreover, rs738409 individuals also had higher plasma alanine aminotransferase (ALT), aspartate aminotransferase (AST), ferritin, and histological fibrosis stages. Jain et al. 37 compared 218 obese Asian Indian adolescents with 86 healthy lean people without fatty liver and found that rs738409 C>G of PNPLA3 may be closely related to obesity and NAFLD in Asian Indian adolescents. Similar experiments were conducted in America 38, Italy 39, and Malaysia 40, and their results were similar.

Sookoian et al. 41 started to study whether PNPLA3 I148M was related to the severity of NAFLD in 2011. Through histological evaluation, they found that PNPLA3 I148M and NAFLD severity had a strong positive correlation that was independent of BMI, sex, age and insulin sensitivity. Recently, Arida et al. 42 performed NAFLD liver fat score and fibrosis score on 13,298 adults without viral hepatitis who had fasted for at least 4 hours. The results showed that patients with PNPLA3 I148M genotype were more likely to have liver fat and fibrosis, and have a higher mortality rate from liver disease. Studies have also shown that NAFLD patients with the PNPLA3 rs738409 G allele carrying the PNPLA3 148M variant have a subtle response to treatment involving dipeptidyl peptidase-4 inhibitors, lifestyle changes and bariatric surgery 43. This suggests that the genetic variant PNPLA3 I148M may be used as one of the markers for monitoring NAFLD.

The key mechanism of PNPLA3-148M causing steatosis is very complicated. Generally speaking, it is related to the relative or absolute increase of TG accumulation in the liver. The first mechanism is related to the lipase activity of PNPLA3. Studies have shown that the PNPLA3-148M variant has reduced TG hydrolase activity and increased TG accumulation in the liver. Huang et al. 20 found that the mutation of PNPLA3 at position 148 (PNPLA3 I148M) would greatly reduce the activity of triacylglycerols (TAGs) and diacyl-glycerol (DAG) hydrolases. Other studies suggested that the accumulation of TAG in the liver of PNPLA3 I148M transgenic mice was not only an increase in TAG synthesis 44. The I148M variant can promote liver steatosis by changing the liver TAG-fatty acid profile and increasing fatty acid formation 44. He et al. 11 found through *in vitro* experiments that PNPLA3-I148M lost the activity of hydrolyzing triglycerides, causing TGs to accumulate in the liver and gradually form fatty degeneration. In several other studies, it was also found that overexpression of PNPLA3-148M in mice and humans could promote the occurrence of steatosis 45, 46. The second mechanism is that PNPLA3-148M will increase the activity of LPAAT to promote the synthesis of TG. Kumari et al. 27 found that the I148M variant had higher LPAAT activity than the wild type. It is worth noting that the LPAAT activity of this variant was much higher than the TG hydrolase activity 27. The third mechanism is related to the reduced ubiquitination of I148M. BasuRay et al. 28 found that the main catabolic pathway of PNPLA3 was related to ubiquitination and proteasome degradation. After I148M transgenic mice were treated with the proteasome inhibitor bortezomib, the ubiquitination ratio of PNPLA3 was much lower than that of WT mice 28. They proposed that PNPLA3-148M can avoid ubiquitination and proteasome degradation, resulting in the accumulation of PNPLA3 in the liver and the reduction of TG mobilization in LDs 28, 47. In short, PNPLA3 I148M, as a high-risk factor for NAFLD, will be a research focus on the treatment of NAFLD in the future.

### PNPLA3 and liver fibrosis

Fibrosis is the end result of many inflammatory and tissue repair reactions, as well as most chronic inflammatory diseases 48, manifested by extracellular Matrix 49. The causes of liver fibrosis include alcoholic liver injury (ALD), NAFLD and hepatitis.

ALD is very common in liver disease. Alcoholic fatty liver (AFL) can develop into alcoholic steatohepatitis (ASH), which is a disease with inflammatory changes in the liver. The long-term existence of ASH will eventually lead to fibrosis and cirrhosis, and even develop into hepatocellular carcinoma (HCC) 50. Kolla et al. 51 conducted a case-control study on patients of European ancestry with a history of ALD, and genotyping single pairs of nucleotide polymorphisms (SNPs), and determined that PNPLA3 SNP rs738409 has a strong correlation between the occurrences of ALD. Trépo et al. 52 selected 328 healthy people and 330 ALD patients in the rs738409 polymorphism genotyping study among European whites, and found that the frequency of rs738409 G alleles in the control group was significantly lower than that of ALD, and rs738409 G alleles genes were also significantly connected with steatosis, fibrosis, and a higher risk of liver cirrhosis. Chamorro et al. 53 conducted a meta-analysis and also confirmed the correlation between the rs738409 G allele and ALD and alcoholic cirrhosis (ALC). Some other studies have also confirmed that PNPLA3 rs738409 was closely related to the occurrence of ALD in whites 54 and Chinese Han males 55.

Hepatic stellate cells (HSCs) are the main participants in liver fibrosis. PNPLA3 is highly expressed in HSCs and participates in HSCs-related retinol metabolism 13, 15. When cells are stimulated by insulin, the expression of PNPLA3 in HSCs increased to promote the release of extracellular retinol 15. PNPLA3 can promote the remodeling of extracellular matrix through this process, thereby preventing the occurrence of fibrosis 15. The PNPLA3 I148M variant's ability to participate in the metabolism of retinol was significantly reduced, which increased the risk of fibrosis 13, 15. In other cell experiments, it was also confirmed that PNPLA3 was essential for activating HSCs. The proliferation and migration of HSCs will be enhanced by the I148M variant, which promotes the development of fibrosis 14. Manchiero et al. 56 tested 290 patients in the clinic hospital of the University of São Paulo and found that the PNPLA3 rs738409 GG genotype plays a major role in the development of hepatitis C (HCV) steatosis and advanced fibrosis. Yasuiet al. 57 also confirmed that this genotype was an independent risk factor for the development of steatosis, severe necrotic inflammation, and advanced liver fibrosis from 276 chronic HCV patients in Japan. In the ordinal logistic regression analysis, the more advanced stage of fibrosis was related to the PNPLA3 rs738409 genotype 58. It is worth noting that in their study, there was no obvious relationship between the PNPLA3 rs738409 genotype and histological necrotizing inflammatory activity 58. Ampuero et al. 59 also proposed that PNPLA3 rs738409 had a greater contribution to steatosis in patients with HCV-1 and IL28B-CT/TT genotypes. Mazo et al. 60 conducted a multi-center cross-sectional study 58. Among them, research on NAFLD patients showed that a CG or GG at rs738409 PNPLA3 makes Brazilians more susceptible to NAFLD. In addition, NASH patients with PNPLA3 GG had more significant increased in liver enzymes and fibrosis. Kupcinskas et al. 61 in the study of Eastern Europeans also proposed that PNPLA3 rs738409 and RNF7 rs16851720 were significantly related to the occurrence of liver fibrosis and cirrhosis. In conclusion, the PNPLA3 rs738409 genotype is significantly related to the progression of ALD, NAFLD and HCV to liver fibrosis, and may be related to the severity of liver fibrosis.

### PNPLA3 and hepatocellular carcinoma

HCC is one of the main causes of death from cancer worldwide 62. HCC caused by viral hepatitis B (HBV) and HCV is the cause of death in developing countries, while HCC caused by large amounts of alcohol intake is the leading cause of death in Europeans and Americans 63-65. A final consequence of chronic hepatitis C (CHC) or chronic hepatitis B (CHB) is sometimes development of HCC, which especially occurs to developing countries (over 80% of HCCs occurs to such regions) 65. In 2011, Valenti et al. 58 proved that the PNPLA3 I148M gene mutation promoted the occurrence of HCC, and they also revealed that this gene mutation can promoted the development of HCV-related cirrhosis into HCC. In 2014, Liu et al. 66 showed that carriers of the PNPLA3 rs738409 C>G polymorphism had a higher risk of HCC. In the meta-analysis, the PNPLA3 rs738409 C>G polymorphism was found to be significantly associated with an increased risk of HCC, and the PNPLA3 rs738409 polymorphism may be a potential biomarker for HCC in whites 67, 68.

Valenti et al. 69 concluded the PNPLA3 148M allele was associated with a shorter history of liver cirrhosis, advanced liver cirrhosis, and lower differentiation of liver cancer in patients with ALD and NAFLD. And the homozygosity of PNPLA3 148M significantly reduced the survival rate of ALD and NAFLD patients. Nischalke et al. 70 also confirmed that the PNPLA3 148M variant was an important risk factor of the development of alcoholic cirrhosis into HCC. Further, the G/G genotype of PNPLA3 rs738409 SNP was correlated with the proceeding of HCC in patients with non-hepatitis B virus (NBNC), and showed a poor prognosis 71. The same conclusion were obtained in the prospective study of Guyot et al. 72, and older age, higher BMI and men were more likely to develop HCC. Takeuchi et al. 71 also mentioned the correlation between BMI and HCC. Burza et al. 73 also pointed out that the PNPLA3 I148M gene mutation was a risk factor for severely obese people suffering from HCC. This indicates that the PNPLA3 148M gene mutation may not be directly related to the occurrence of tumors. From previous studies, it can be found that the mutation of PNPLA3 148M gene may not be directly related to the occurrence of tumors. PNPLA3 148M gene mutation may increase the probability of HCC by increasing steatosis, promoting fibrosis, etc. 11, 14, 20, 71, 73.

## PNPLA3 and other diseases

### PNPLA3 and type 2 diabetes mellitus

Diabetes is one of the common chronic diseases, which is related to decreased insulin secretion and weakened or disappeared insulin action 74. Type 2 diabetes (T2DM) patients account for 90% to 95% of all diabetes patients, and the number is still increasing, which may be related to people's lifestyle changes. The occurrence of T2DM is not only related to environmental factors, but also related to genetic factors. The mutation of PNPLA3 as a genetic variation also has an important impact on the occurrence and development of T2DM 75, 76.

Bellan et al. suggested that T2DM patients with rs738409 mutant may be more likely to develop NAFLD 77. Xia et al. 78 found that among participants with poor metabolism, the PNPLA3 genotype played a subtle role in weight changes and exacerbated steatosis. It is worth noting that this genotype was found in this study to reduce the risk of T2DM. Petit et al. 79 also showed that the increase in liver fat contented in T2DM patients was related to PNPLA3 rs738409, and the liver steatosis of minor G allele carriers may be more obvious. Machado et al. 80 showed that the PNPLA3 rs738409c>G polymorphism seemed to be related to the development of complications of T2DM, and the GG genotype was more closely related to the occurrence of liver fibrosis, and both CG and GG genotypes were associated with cardiovascular complications related. It is worth noting that patients with G alleles have better blood glucose control 80. It is also mentioned in other studies that PNPLA3 SNP rs738409 has no obvious correlation with changes in ALT, AST, ALP and other indicators 81. These results suggest that the T2DM-related complications caused by this genetic mutation may not have much to do with metabolism in the body.

In some studies, the relationship between PNPLA3 SNP rs738409 and insulin resistance was also discussed. It was found that although this gene is not only not related to insulin sensitivity, it is also not related to the levels of liver enzymes, high-sensitivity c-reactive protein and fetuin-A, suggested that PNPLA3 SNP rs738409 mutation did not cause insulin resistance 35, 82, 83. Previous experiments have shown that PNPLA3 I148M does not seem to cause high liver fat content and insulin resistance 35, 82, and recent research showed a deeper relationship. Barata et al. 84 found through meta-analysis that reducing insulin resistance and increasing insulin levels in non-diabetic patients carrying this gene can improve liver steatosis.

From the above results, PNPLA3 I148M was found to be not directly related to the occurrence of diabetes, and the development of diabetes-related complications will be promoted by it. This association is still related to the aforementioned PNPLA3 I148M variant promoting lipid accumulation.

### PNPLA3 and coronary artery disease

Cardiovascular atherosclerosis (CAD) causes a large proportion of deaths in both developing and developed countries 85. Many factors are related to the increase in the incidence of adverse events. A large number of studies have found that in addition to showing liver pathological manifestations, NAFLD also increases the risk of cardiovascular diseases including CAD 86, 87.

According to previous research, PNPLA3 was a factor that was significantly related to susceptibility to NAFLD, and CAD was the main contributor to the death of NAFLD patients. Posadas-Sánchez et al. 88 evaluated whether the PNPLA3 gene can be used as an early diagnostic marker for early coronary artery disease (pCAD) and cardiovascular risk factors. They used the exonuclease TaqMan assay to test the genotype polymorphisms of 2572 subjects. The results showed that PNPLA3 (rs738409) polymorphism could be used as a marker to help diagnose pCAD. Machado et al. 80 performed an analysis on 303 patients with diabetes mellitus and NAFLD (118 males, mean age 59±9.5 years). For patients with diabetes and NAFLD, on the one hand, the PNPLA3 gene rs738409 C>G polymorphism increased the risk of cardiovascular disease, on the other hand, it can play a beneficial role in blood glucose control. Petta et al. 89 also found that among NAFLD patients, patients with PNPLA3 rs738409 GG genotype were more likely to develop carotid atherosclerosis. In summary, NAFLD patients with PNPLA3 rs738409 variants are at greater risk of developing CAD. Brouwers et al. 90 concluded that the NAFLD susceptibility gene (PNPLA3 I148M) itself did not cause CAD, and the relationship between PNPLA3 and CAD was mainly through plasma lipids. In short, the PNPLA3 I148M variant can promote the development of NAFLD, thereby increasing the risk of CAD in patients.

### PNPLA3 and kidney disease

Chronic kidney disease (CKD), as usual chronic disease, affects approximately 8-16% of adults worldwide, and the prevalence increases sharply with age 91. At present, with the accumulation of research on PNPLA3, NAFLD is more closely related to kidney function, and the growth of chronic kidney disease (CKD) may also be related to PNPLA3 92, 93.

Sun et al. 94 attempted to explore which biomarkers related to renal tubular injury (RTI) and glomerular function changes were affected by the PNPLA3 genotype. Their patients were those who had NAFLD with either normal alanine aminotransferase levels (nALT) or abnormal alanine aminotransferase levels (abnALT). They studied 217 patients with histologically confirmed NAFLD, 75 of whom had sustained nALT levels (three months below the normal upper limit). The results showed that the nALT patient groups had higher albuminuria than the abnALT group and had a higher prevalence of CKD. They concluded that adult patients with the PNPLA3 rs738409G allele, NAFLD, and persistent nALT are more likely to develop early glomerular and tubular damage. The molecular mechanisms remain unknown. Mantovani et al. 95 studied 101 postmenopausal white women with T2DM and found that the rs738409 G/G genotype of the PNPLA3 gene was connected with a lower level of e-GFRCKD-EPI and a higher risk of CKD. There was no obvious correlation with NAFLD and other risk factors, which has also been confirmed in other studies 96. In addition, studies have shown that this genotype was related to the decline in renal function of the elderly in Japan 97.

In addition, Targher et al. 98 performed a similar study on Caucasian children with biopsy-proven NAFLD, and showed for the first time that decreased e-GFR and increased 24-hour urinary protein excretion were strongly associated with the G allele of rs738409. Costanzo et al. 99 also obtained the same result through a study of 230 obese children and adolescents, that is, the PNPLA3 rs738409 variant increases the risk of impaired renal function in children with NAFLD. Regarding the cause of renal damage, research speculated that it may be related to retinol metabolism, lipid metabolism and podocyte damage 96. NAFLD is considered to be an independent risk factor for CKD. Based on the above research, it is speculated that the PNPLA3 I148M variant promotes the development of CKD is still related to the susceptibility of NAFLD.

## Further challenges

The fact that PNPLA3 is a factor that is significantly related to the susceptibility of NAFLD is now well established 100-102. Thus, some researchers think they can study precision medicine in NAFLD with PNPLA3 as a therapeutic target 103. Although the PNPLA3 I148M in the pathology of fatty liver disease is well understood, further research is clearly needed to further expand the molecular mechanisms associated with these new findings and to further validate these results. PNPLA3 I148M is closely related to liver disease and its complications, and according to the current research, it is mainly related to the accumulation of lipids in the liver. But its specific mechanism also needs further research to clarify.

The recent research in murine models shows that antisense oligonucleotide therapy for PNPLA3 can delay the progress of NAFLD and improve liver fibrosis. Importantly, it can also inhibit the expression of PNPLA3 148M 104. Although this experiment was conducted in mice, in future, it may also open to a precision medicine approach from NASH.

Studies of inhibitors or agonists of PNPLA3 are relatively few, and even now, people understand that there is a strong association between PNPLA3 and those diseases, thus research on inhibitors or agonists of PNPLA3 and its variant could be further investigated in the next step.

## Figures and Tables

**Figure 2:**
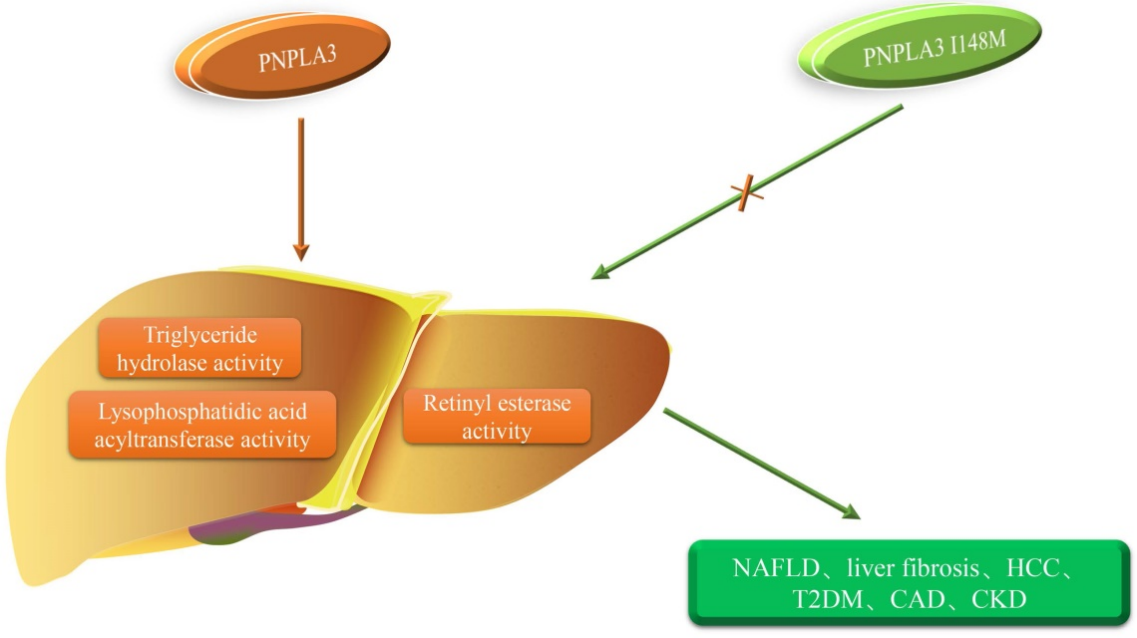
** PNPLA3 I148M and the occurrence of diseases.** The orange arrow represents the role of PNPLA3, and the green arrow represents the role of PNPLA3 I148M. PNPLA3, patatin-like phospholipase domain-containing 3 gene; NAFLD, non-alcoholic fatty liver disease; HCC, hepatocellular carcinoma;T2DM, type 2 diabetes mellitus; CAD, coronary artery disease; CKD, chronic kidney disease.

**Table 1:** Study on the related mechanism of PNPLA3 and its variants in promoting NAFLD

Key Finding	Year	Reference
Found a non-synonymous variant of PNPLA3, PNPLA3 (rs738409, I148M).	2008	35
PNPLA3 deletion is not associated with fatty liver, lipid homeostasis and insulin resistance in mice.	2010, 2011	25,26
PNPLA3 I148M significantly reduces the activity of TAGs and DAG hydrolase and changes the liver TAG-fatty acid profile.	2012	44
The PNPLA3-I148M variant loses its triglyceride hydrolysis activity.	2010, 2015, 2016	11,45,46
The I148M variant showed increased LPAAT activity leading to an increase in cellular lipid accumulation.	2012	27
PNPLA3- I148M can avoid ubiquitination and proteasome degradation, resulting in a decrease in TG mobilization in LDs.	2017	28
PNPLA3 promotes the transfer of vLCPUFAs from TG to PLs in liver lipid droplets, and PNPLA3-I148M enhances this effect.	2018	21

PNPLA3, patatin-like phospholipase domain-containing protein 3; TAG, triacylglyceride; DAG, diacylglycerol; LPAAT, lysophophosphatidic acid acyltransferase; TG, triacylglycerol; LDs, lipid droplets; vLCPUFAs, very long-chain polyunsaturated fatty acids; PLs, phospholipids.

## Abbreviations

PNPLA3: patatin-like phospholipase domain-containing 3 gene
TG: triglyceride
PL: phospholipid
BMI: body mass index
LPAAT: lysophosphatidic acid acyltransferase activity
NAFLD: non-alcoholic fatty liver disease
NASH: non-alcoholic steatohepatitis
ALT: alanine transaminase
AST: aspartate transaminase
CGI-58: comparative gene identification-58
ATGL: adipose triglyceride lipase
ALD: alcoholic liver injury
CHC: chronic hepatitis C
CHB: chronic hepatitis B
HCC: hepatocellular carcinoma
SNPs: single pairs of nucleotide polymorphisms
HCV: hepatitis C virus
CBM: Chinese Biomedical Database
T2DM: type 2 diabetes mellitus
CAD: coronary artery disease
CKD: chronic kidney disease
RTI: renal tubular injury
nALT: normal alanine aminotransaminase levels
abnALT: abnormal alanine aminotransaminase levels
e-GFR: estimated Glomerular Filtration Rate
